# Central nervous system effects of tumor necrosis factor alpha blockade

**DOI:** 10.1186/1479-5876-9-S2-I2

**Published:** 2011-11-23

**Authors:** Georg Schett

**Affiliations:** 1 Dept. of Internal Medicine 3, Erlangen, Germany

## 

The therapeutic success of TNFa blockade in RA is unique and has been largely considered to result from rapid and efficient neutralization of joint inflammation based on breakdown of the inflammatory cytokine network in the affected joint, which results in an improvement of the signs and symptoms of the disease (4). It has, however, always been stunning, how fast the blockade of TNFa improves the patient condition, in particular since diseases like RA are highly chronic, building up a vast amount of inflammatory tissue and leading to irreversible damage of the cartilage and the bone. Thus, rapid resolution of this highly organized inflammatory tissue and/or tissue damage is very unlikely to explain the fast effect of TNFa blockade. There has always been a gap in understanding of how tumor necrosis factor alpha (TNFa) blockade affects the disease state of arthritis patients so rapidly, considering that diseases like rheumatoid arthritis are very chronic conditions. We thus hypothesized that blockade of TNFa acts through the central nervous system (CNS) before directly affecting joint inflammation. By use of functional magnetic resonance imaging (fMRI), we demonstrate that within 24 hours after neutralization of TNFa nociceptive CNS activity in the thalamus and somatosensoric cortex but also the activation of the limbic system is blocked. Brain areas showing blood oxygen level dependent (BOLD) signals, a validated method to assess neuronal activity elicited by pain, were significantly reduced as early as 24 hours after initiation of TNFa blockade. In contrast, clinical and laboratory markers of inflammation such as joint swelling and acute phase reactants were not affected by TNFa blockade at these early time points. Moreover, arthritic mice overexpressing human TNFa showed an altered pain behavior and a more intensive, widespread and prolonged brain activity upon nociceptive stimuli as compared to wild-type mice. Similar to humans, these changes as well as the rewiring of CNS activity resulting in tight clustering in the thalamus were rapidly reversed after neutralization of TNFa. These results suggest that neutralization of TNFa affects nociceptive brain activity in the context of arthritis, long before it achieves anti-inflammatory effects in the joints.

